# The silent risk: cardiometabolic burden and etiology of young stroke in a South Asian migrant cohort, United Arab Emirates

**DOI:** 10.3389/fneur.2026.1940843

**Published:** 2026-09-17

**Authors:** Mohammed AlOmar, Basma AlOmar, Lamees Alsalehi, Abdulrahman Said Adan, Aya Alsayyad, Ammar Fadhil AlOmar

**Affiliations:** 1 Internal Medicine, Ibrahim Bin Hamad Obaidullah Hospital, Ras Al Khaimah, United Arab Emirates; 2 Emergency Department, Ibrahim Bin Hamad Obaidullah Hospital, Ras Al Khaimah, United Arab Emirates; 3 Department of Neurology, Ibrahim Bin Hamad Obaidullah Hospital, Ras Al Khaimah, United Arab Emirates

**Keywords:** cerebral infarction, delayed diagnosis, diabetes mellitus, dyslipidemias, hypertension, intracranial hemorrhages, primary health care, sinus thrombosis

## Abstract

Young-adult stroke in the Arabian Gulf remains poorly characterized, despite a resident population dominated by South Asian labor migrants in whom cardiometabolic disease is often under-diagnosed. We therefore aimed to characterize the etiology and vascular risk-factor burden of young stroke in a single-center United Arab Emirates cohort, testing whether the recorded risk profile reflects true prevalence or diagnostic gaps. In this retrospective cohort study, we reviewed all patients aged 18–49 years with radiologically confirmed stroke admitted to a secondary-care hospital in Ras Al Khaimah between January 2020 and March 2026, applying adjudicated subtype classification, and used a pre-specified multivariable model to identify factors associated with ischemic presentation. Of 421 patients screened, 314 met criteria (mean age 41.0 years; 81.2% male; 65.6% South Asian). Objectively measured risk far exceeded documented history: only 49.0% had recorded hypertension, yet 74.5% had elevated admission blood pressure; dyslipidemia was documented in 15.9%, yet an abnormal lipid profile was found in 73.9% of those tested; and 29% of all diabetes was first diagnosed at the stroke admission. Small-vessel occlusion was the leading determined ischemic subtype, and diabetes was the only factor independently associated with ischemic stroke (adjusted OR 2.34, 95% CI 1.35–4.07). South Asian patients differed chiefly by sex and ischemic predominance rather than independent metabolic excess, indicating that ethnicity defined the exposed population while diabetes and hypertension were the operative mechanisms. We conclude that, in this workforce, the low recorded prevalence of vascular risk reflects widespread under-diagnosis, not low true prevalence—stroke was frequently the first presentation of unmanaged cardiometabolic disease, identifying accessible primary care and workplace screening as concrete prevention priorities.

## Introduction

Stroke in young adults, conventionally defined as occurring between 18 and 49 years of age, accounts for a small but rising fraction of all strokes, and carries disproportionate societal cost because it strikes individuals during their most economically productive years (1, 2). The risk factors, etiologies, and outcomes of stroke in the young differ substantially from those in older patients, and they vary considerably between regions and ethnic groups (3, 4). Most of the available evidence derives from European and North American registries, whose populations differ profoundly from those of the Arabian Gulf (1, 2).

Although stroke remains predominantly a disease of later life, its burden among young adults is substantial and increasing. Stroke was the third most common cause of death globally in 2021, accounting for 10.7% of all deaths, and the contribution of metabolic risk factors to stroke burden rose over the preceding three decades (5). Against a background of falling age-standardized rates overall, incidence among adults aged 18 to 50 years has risen in several settings, a divergence attributed in part to the increasing prevalence of hypertension, diabetes, obesity, and smoking in younger cohorts (6, 7). This trend is pronounced in the Middle East and North Africa, where pooled prevalence of young stroke more than tripled between studies conducted before and after 2015, rising from 0.022 to 0.072% (8). The clinical and societal consequences are disproportionate to case numbers. Young survivors face decades of life after stroke, with elevated long-term mortality and recurrence risk relative to age-matched peers, and the resulting demand for long-term care poses a sustained challenge to health systems (6). Vocational outcomes are correspondingly poor: a substantial minority of working-age survivors do not return to employment, with consequent loss of productivity and of individual economic independence (9). Where the affected population is a migrant workforce, this loss extends beyond the individual, since remitted earnings frequently support dependents in the country of origin (10). These considerations make the recognition and control of modifiable vascular risk factors in young adults *a priori*ty that is at once clinical and economic.

The United Arab Emirates (UAE) presents an unusual epidemiological setting. Its resident population is dominated by expatriate labor migrants, the majority of whom are young men from South Asia. This demographic structure, combined with a regional burden of cardiometabolic disease driven by rapid urbanization, dietary transition, and sedentary lifestyle, may produce a young-stroke profile distinct from those described elsewhere. Yet published data on stroke in young adults from the Gulf, and from the UAE specifically, remain limited, and existing regional reports are often pooled across wide age ranges or multiple heterogeneous centers (11, 12).

We therefore conducted a retrospective cohort study of young adults admitted with stroke to a single secondary-care center in the northern UAE, applying consistent, adjudicated subtype classification across the full spectrum of cerebrovascular disease, ischemic stroke, intracerebral hemorrhage, subarachnoid hemorrhage, and cerebral venous sinus thrombosis. Our aims were to describe the etiology and vascular risk-factor profile of young stroke in this population, to examine how that profile differs by stroke subtype and across nationality groups, and to identify factors independently associated with ischemic presentation.

## Materials and methods

### Study design and setting

We performed a single-center, retrospective chart review of consecutive patients admitted with acute stroke to Ibrahim Bin Hamad Obaidullah Hospital (IBHOH), a secondary-care hospital in Ras Al Khaimah, United Arab Emirates, between 1 January 2020 and 31 March 2026. IBHOH is a medical hospital without on-site surgical neurovascular or neurosurgical services; cases requiring surgical or interventional management are referred to its partner surgical facility, Saqr Hospital. This referral arrangement has implications for the ascertainment of surgically managed hemorrhagic strokes, as discussed in the limitations. The study was reported in accordance with the STROBE guidance for observational studies (13). A companion analysis of the same registry, addressing diagnostic workup adequacy and quality of care rather than etiology, is in preparation; that analysis deliberately applies a broader cohort definition, retaining clinically diagnosed but radiologically unconfirmed strokes because confirmation status is itself one of its outcomes. All data were de-identified prior to analysis.

### Participants and case definition

#### Inclusion criteria

Patients were eligible for inclusion if they met all of the following criteria: (i) aged 18 to 49 years at the time of admission; (ii) a diagnosis of stroke confirmed by neuroimaging (computed tomography and/or magnetic resonance imaging); and (iii) an event classified as acute ischemic stroke (AIS), intracerebral hemorrhage (ICH), subarachnoid hemorrhage (SAH), or cerebral venous sinus thrombosis (CVST). Repeat stroke events in the same patient occurring as distinct admissions were retained as separate events.

#### Exclusion criteria

Patients were excluded if any of the following applied: (i) absence of radiological confirmation of stroke (*n* = 68); (ii) a final diagnosis of transient ischemic attack or stroke mimic (*n* = 30); (iii) in-hospital stroke occurring in a patient admitted for another indication (*n* = 6), since presentation and recognition pathways differ systematically from community-onset stroke; (iv) duplicate records for a single admission (*n* = 2); and (v) traumatic intracranial hemorrhage (*n* = 1). Of 421 patients screened, 107 were excluded on these grounds, leaving a final analytic cohort of 314. The selection of the analytic cohort is summarized in Figure 1.

**Figure 1:**
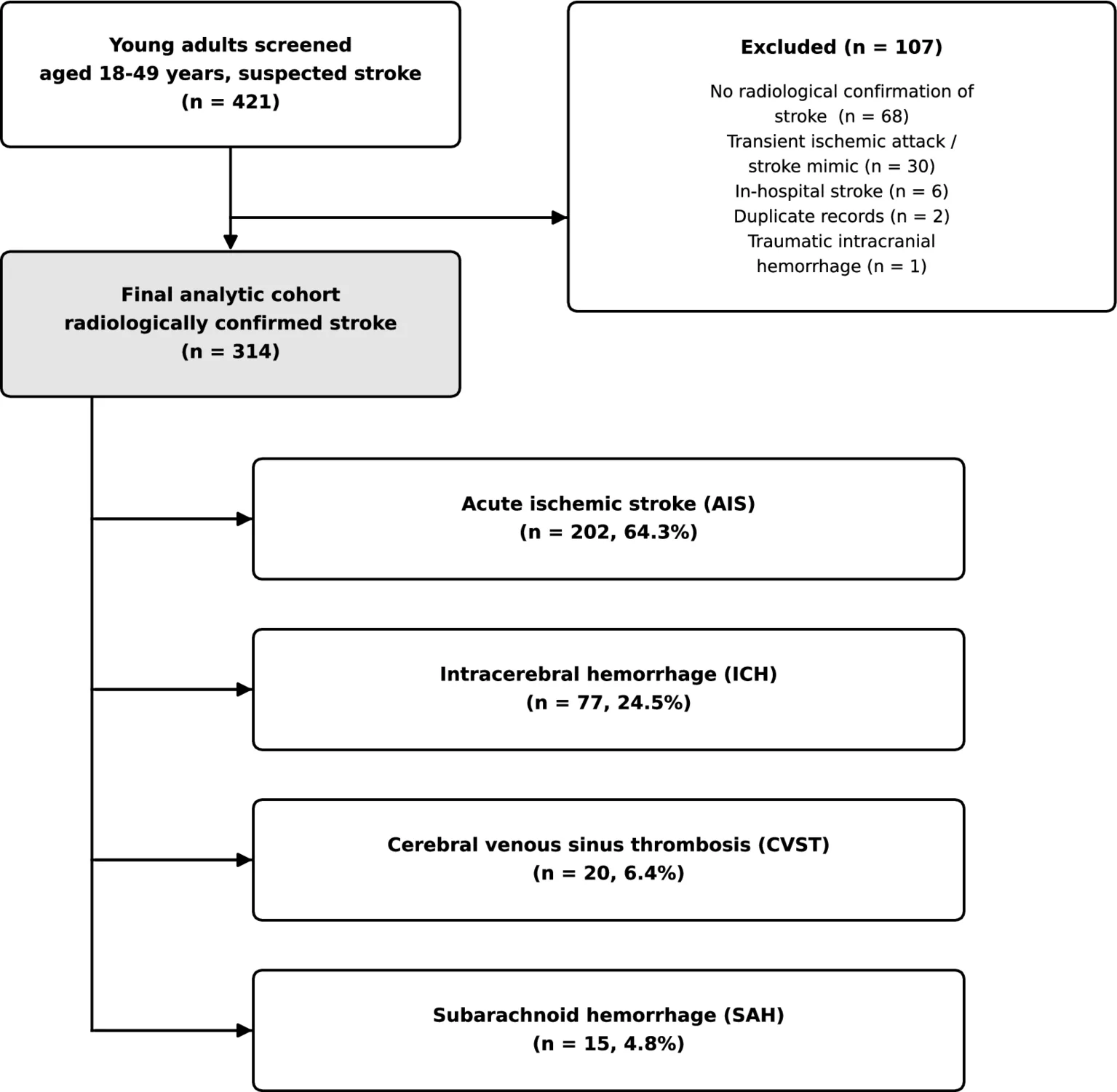
Selection of the analytic cohort and distribution by stroke subtype (STROBE-style flow diagram).

The disposition of other secondary and structural causes was as follows. Hemorrhage attributable to an underlying neoplasm, and hemorrhagic transformation of an ischemic infarct, were not eligible for inclusion as primary hemorrhagic events. Structural causes of intracerebral hemorrhage, including arteriovenous and cavernous malformations, were retained and classified within the SMASH-U structural-lesion category rather than excluded. Both aneurysmal and non-aneurysmal subarachnoid hemorrhage were retained and analyzed within the separately classified SAH stratum, and cerebral venous sinus thrombosis was retained as a standalone category.

### Classification of stroke etiology

Ischemic strokes were classified according to the Trial of Org 10,172 in Acute Stroke Treatment (TOAST) criteria into large-artery atherosclerosis, cardioembolism, small-vessel occlusion, other determined etiology, and undetermined etiology (14). Large-artery atherosclerosis required corroborating vascular imaging; artery-to-artery embolism from non-stenotic plaque was classified as other determined etiology rather than large-artery atherosclerosis. Undetermined strokes were sub-classified by the reason classification could not be completed (incomplete cardiac, non-cardiac, or multiple workup) or by the presence of competing etiologies. Intracerebral hemorrhage was classified by the SMASH-U system (structural lesion, medication, cerebral amyloid angiopathy, systemic/other disease, hypertension, undetermined) (15). Subarachnoid hemorrhage was categorized as aneurysmal or non-aneurysmal. CVST was treated as a distinct, non-arterial category and was not subjected to TOAST classification. All classifications were performed by a single adjudicator applying consistent criteria, with full reasoning documented for reproducibility.

The four subtypes were classified independently and were never pooled for etiologic analysis. TOAST was applied only to ischemic stroke and SMASH-U only to intracerebral hemorrhage. Subarachnoid hemorrhage was classified separately as aneurysmal or non-aneurysmal, and cerebral venous sinus thrombosis was treated as a standalone category outside both frameworks. Anatomical location of intracerebral hemorrhage was recorded separately from etiology and grouped as deep, lobar, or infratentorial.

### Variables and definitions

Vascular risk factors were defined as pre-existing diagnoses combined, where appropriate, with confirmatory testing performed during admission. Diabetes was defined as chronic diabetes throughout: a patient was classified as diabetic if previously diagnosed or if glycated hemoglobin (HbA1c) was ≥6.5% during admission. Because HbA1c reflects glycaemia over the preceding several weeks, an elevated value is confirmatory of chronic, pre-existing disease rather than of an acute disturbance; the 30 patients first diagnosed on this basis were therefore classified as chronic diabetics, and admission plasma glucose alone was not used for diagnosis. Hypertension was handled differently, precisely because a single admission reading cannot establish chronicity: acute stroke triggers a transient pressor response. For descriptive purposes (Table 1) we therefore report documented prior history of hypertension and, as a separate variable, elevated admission blood pressure (systolic ≥140 mmHg and/or diastolic ≥90 mmHg at presentation; available for all patients). For comparative and regression analyzes a single composite hypertension variable was used, defined as documented history of hypertension OR elevated admission blood pressure ≥140/90 mmHg (248 patients, 79.0%); this definition is explicit and reproducible, and we acknowledge that, by including admission readings, it may incorporate some transient pressor responses in addition to true chronic hypertension. Dyslipidemia was captured both as a documented historical diagnosis and as an abnormal admission lipid profile derived from measured lipid panels (elevated total cholesterol, LDL cholesterol, or triglycerides, or low HDL cholesterol by standard thresholds), reported among patients in whom lipids were measured. Stroke severity was captured as a categorical National Institutes of Health Stroke Scale (NIHSS) band for ischemic strokes only, as the NIHSS is validated for ischemic stroke; it was not applied to hemorrhagic or venous events.

**Table 1:** Baseline characteriztics of young adults with stroke (18–49 years), stratified by stroke subtype.

Characteristics	AIS (*n* = 202)	ICH (*n* = 77)	SAH (*n* = 15)	CVST (*n* = 20)	Total (*n* = 314)	*p*
Demographics						
Age, years, mean ± SD	41.3 ± 5.8	41.3 ± 6.0	37.9 ± 6.2	39.1 ± 5.8	41.0 ± 5.9	0.064^h^
Male sex	172 (85.1)	61 (79.2)	10 (66.7)	12 (60.0)	255 (81.2)	0.017
Nationality / region of origin						
Emirati / UAE national	7 (3.5)	3 (3.9)	0 (0)	2 (10.0)	12 (3.8)	
Other Arab / MENA	20 (9.9)	4 (5.2)	4 (26.7)	6 (30.0)	34 (10.8)	
South Asian	144 (71.3)	48 (62.3)	6 (40.0)	8 (40.0)	206 (65.6)	
Other	31 (15.3)	22 (28.6)	5 (33.3)	4 (20.0)	62 (19.7)	
Vascular risk factors						
Known hypertension (history)^d^	99 (49.0)	49 (63.6)	6 (40.0)	0 (0)	154 (49.0)	<0.001^i^
Elevated admission BP ≥ 140/90^e^	151 (74.8)	71 (92.2)	8 (53.3)	4 (20.0)	234 (74.5)	
Diabetes mellitus^d^	80 (39.6)	16 (20.8)	4 (26.7)	3 (15.0)	103 (32.8)	0.006
Dyslipidemia (history)	38 (18.8)	9 (11.7)	2 (13.3)	1 (5.0)	50 (15.9)	0.246
Abnormal admission lipid profile^f^	136/179 (76.0)	34/52 (65.4)	5/7 (71.4)	9/11 (81.8)	184/249 (73.9)	
Smoking^a^	53/161 (32.9)	9/46 (19.6)	2/10 (20.0)	2/15 (13.3)	66/232 (28.4)	
Ischemic heart disease	14 (6.9)	1 (1.3)	0 (0)	0 (0)	15 (4.8)	
Prior stroke	13 (6.4)	6 (7.8)	0 (0)	0 (0)	19 (6.1)	
Prior TIA	5 (2.5)	0 (0)	0 (0)	0 (0)	5 (1.6)	
Atrial fibrillation	3 (1.5)	0 (0)	0 (0)	0 (0)	3 (1.0)	
BMI, kg/m^2^, mean ± SD^b^	27.7 ± 5.0	27.5 ± 5.4	25.8 ± 4.5	26.5 ± 4.0	27.5 ± 5.1	
Admission glucose, mmol/L, median (IQR)^g^	7.5 (6.0–12.0)	7.3 (6.4–9.2)	7.7 (6.0–9.5)	6.2 (5.3–7.6)	7.4 (6.0–10.7)	
Stroke severity (NIHSS band), ischemic stroke only^c^						
Minor (0–4)	101 (50.0)	—	—	—	—	
Moderate (5–15)	89 (44.1)	—	—	—	—	
Severe (≥16)	12 (5.9)	—	—	—	—	

Values are n (%) unless otherwise stated. Percentages may not sum to 100 due to rounding. *p*-values compare the distribution across the four stroke subtypes (Fisher’s exact test, or Kruskal–Wallis for age); only pre-specified comparisons are reported. Diabetes is reported consistently as chronic diabetes throughout. For hypertension, this table reports documented history and elevated admission blood pressure separately; the composite hypertension variable (documented history OR admission BP ≥140/90 mmHg; 248, 79.0%) was used in the comparative and regression analyses. AIS, acute ischemic stroke; BMI, body mass index; CVST, cerebral venous sinus thrombosis; ICH, intracerebral hemorrhage; MENA, Middle East and North Africa; NIHSS, National Institutes of Health Stroke Scale; SAH, subarachnoid hemorrhage; SD, standard deviation; TIA, transient ischemic attack.

a Smoking reported as events/documented (%); status missing in 82 patients.

b BMI available in 296 patients.

c NIHSS reported for the AIS subgroup only.

d Diabetes reflects chronic diabetes (prior diagnosis or HbA1c ≥ 6.5% at admission, which is confirmatory of chronicity); 30 of the 103 cases were first diagnosed at this admission. Hypertension is reported as documented history only, separately from elevated admission blood pressure, because the acute pressor response of stroke precludes a single-admission reading from confirming chronicity.

e Elevated admission blood pressure defined as systolic ≥140 and/or diastolic ≥90 mmHg at presentation; available for all patients.

f Abnormal admission lipid profile reported as a proportion of patients with lipids measured (*n* = 249); 65 were not tested.

g Admission glucose reported as a measured admission value only; it was not used to diagnose diabetes, given that acute stroke can cause stress hyperglycemia in non-diabetic patients.

h Kruskal–Wallis test.

i The across-subtype *p*-value for hypertension refers to the composite hypertension variable (documented history OR admission BP ≥140/90 mmHg); the rows above show the two components separately.

Nationality was grouped into four categories for analysis: Emirati/UAE national, other Arab/Middle East and North Africa (MENA), South Asian (India, Pakistan, Bangladesh, Sri Lanka, Nepal, Afghanistan), and other. Functional outcome was assessed by the modified Rankin Scale (mRS) at hospital discharge and dichotomised as favorable (0–2) or unfavorable (3–5), with death (mRS 6) reported separately.

### Statistical analysis

Analyses followed a pre-specified sequence of description, comparison, and modeling. Categorical variables were summarized as counts and percentages. Normality of continuous variables was assessed by the Shapiro–Wilk test; all departed significantly from normality (all *p* < 0.001; Supplementary Table S3), and continuous variables were therefore summarized as median with interquartile range, with mean ± standard deviation additionally reported for age to permit comparison with published cohorts. Between-group comparisons used the chi-square test, or Fisher’s exact test where any expected cell count was below five, given the small SAH and CVST subgroups. Continuous variables were compared by the Mann–Whitney U or Kruskal–Wallis test as appropriate. Comparisons were pre-specified; no multiplicity correction was applied, and exact *p*-values are reported. A single pre-specified binary logistic regression model examined factors independently associated with ischemic (versus non-ischemic) stroke, entering age, sex, South Asian origin, diabetes, and hypertension simultaneously; adjusted odds ratios (OR) with 95% confidence intervals (CI) are reported. Analyses were performed in jamovi (version 2.7.37; The jamovi Project), which uses R (version 4.6.1), with two-sided *p* < 0.05 considered significant.

No a priori sample-size calculation was performed. The study used consecutive census sampling: all eligible patients presenting over the 75-month study period were included, so the sample size was determined by the study window rather than selected. *Post-hoc* precision was therefore assessed instead; with *n* = 314 the half-width of the 95% confidence interval for the principal proportions ranged from 4.0 to 5.5 percentage points. The multivariable model included 202 events and 112 non-events, giving 22.4 events per variable for the five predictors entered.

A sensitivity analysis restricted to ischemic stroke and intracerebral hemorrhage (*n* = 279), excluding subarachnoid hemorrhage and cerebral venous sinus thrombosis, was performed to confirm that retention of these subtypes did not influence the principal findings (Supplementary Table S3).

## Results

### Cohort and baseline characteristics

Of 421 young adults screened, 107 were excluded, leaving a final analytic cohort of 314 patients; the exclusion cascade and subtype breakdown are shown in Figure 1. Ischemic stroke accounted for the majority of cases, with smaller numbers of intracerebral hemorrhage, subarachnoid hemorrhage, and cerebral venous sinus thrombosis.

Baseline characteristics, stratified by stroke subtype, are presented in Table 1. The cohort was young (median age 42 years, interquartile range 38–46; mean 41.0 ± 5.9), predominantly male, and predominantly of South Asian origin, reflecting the underlying expatriate-worker demography of the region. Hypertension and diabetes were the most frequent vascular risk factors, and their distribution differed conspicuously across subtypes, a pattern examined formally below. Diabetes was present in 32.8% of patients, of whom 30 were first identified through an admission HbA1c ≥ 6.5% and reclassified as chronic diabetics. Two features of the admission data are notable. First, while a documented hypertension history was present in 49.0% of patients, elevated admission blood pressure (≥140/90 mmHg) was far more common (74.5%), and mean admission pressure was highest in intracerebral hemorrhage (188/115 mmHg) and lowest in cerebral venous sinus thrombosis (123/81 mmHg). Second, although dyslipidemia was recorded as a historical diagnosis in only 15.9% of patients, an abnormal admission lipid profile was present in 184 of 249 patients tested (73.9%), a marked discrepancy examined in the Discussion.

The full breakdown of demographics, subtypes, and risk factors across the four nationality groups is provided in Supplementary Table S1. South Asian patients formed the largest group and were almost exclusively male; cell counts in the Emirati group were small and the corresponding proportions should be interpreted with caution.

### Stroke etiology

Etiologic classification by subtype is detailed in Table 2. Among ischemic strokes, small-vessel occlusion was the most frequent determined mechanism, and large-artery atherosclerosis was comparatively uncommon. A large undetermined fraction was, on sub-classification, attributable chiefly to incomplete cardiac and incomplete multiple workup rather than to true cryptogenic stroke. Intracerebral hemorrhage was overwhelmingly hypertensive, with no case attributable to medication or cerebral amyloid angiopathy, as expected at this age, and most subarachnoid hemorrhages were aneurysmal.

**Table 2:** Stroke etiology by subtype.

Etiological category	n (%)
Ischemic stroke, TOAST classification (*n* = 202)	
Large-artery atherosclerosis	23 (11.4)
Cardioembolism	28 (13.9)
Small-vessel occlusion	55 (27.2)
Other determined etiology	15 (7.4)
Undetermined etiology	81 (40.1)
Incomplete cardiac workup	28 (13.9)
Incomplete non-cardiac workup	22 (10.9)
Incomplete multiple workup	27 (13.4)
Competing etiologies	2 (1.0)
Cryptogenic (complete evaluation, no cause identified)	2 (1.0)
Intracerebral hemorrhage, SMASH-U classification (*n* = 77)	
Hypertension	67 (87.0)
Structural lesion (vascular malformation, tumor)	2 (2.6)
Medication (anticoagulation)	0 (0)
Amyloid angiopathy	0 (0)
Systemic / other disease	1 (1.3)
Undetermined	7 (9.1)
Subarachnoid hemorrhage (*n* = 15)	
Aneurysmal	12 (80)
Non-aneurysmal	3 (20)

Percentages are within-subtype and may not sum to 100 due to rounding. The “other determined etiology” category (*n* = 15) comprised inflammatory or infection-associated arteriopathy (COVID-19–associated, 3; Takayasu arteritis, 2; systemic vasculitis with rheumatoid arthritis, 1; Behçet disease, 1), autoimmune or connective-tissue disease (systemic lupus erythematosus, 1; autoimmune hepatitis, 1; hypergammaglobulinaemia, 1), prothrombotic or embolic sources (subclavian thrombus with thrombophilia, 1; oral contraceptive–associated, 1), and arterial-wall pathology (arterial dissection, 1; aortic dissection with carotid occlusion, 1; carotid plaque embolism, 1). CVST (*n* = 20) is not subjected to TOAST. TOAST, Trial of Org 10,172 in Acute Stroke Treatment.

Intracerebral hemorrhages were predominantly deep in location (54 of 77, 70.1%), with infratentorial hemorrhage in 12 (15.6%) and lobar hemorrhage in 11 (14.3%). Deep hemorrhages comprised putamen (*n* = 37), thalamus (*n* = 15), and caudate (n = 2); infratentorial hemorrhages comprised brainstem (*n* = 9) and cerebellum (*n* = 3). Under SMASH-U, 67 of 77 hemorrhages (87.0%) were attributed to hypertension, 7 (9.1%) were undetermined, 2 (2.6%) were structural, and 1 (1.3%) was attributed to systemic or other disease. Of the 15 subarachnoid hemorrhages, 12 were aneurysmal and 3 non-aneurysmal. The full anatomical and etiologic distribution is given in Supplementary Table S3.

### Presentation, investigations, treatment, and outcomes

Regarding presentation and acute management, patients arrived by private vehicle in 118 cases (37.6%), by emergency medical services in 107 (34.1%), and by inter-facility transfer in 89 (28.3%). A lipid profile was obtained in 249 patients (79.3%) and echocardiography in 106 (33.8%); among ischemic strokes, CT angiography was performed in 120 (59.4%). Intravenous thrombolysis was administered to 51 patients (25.2% of ischemic strokes), with a median door-to-CT time of 26 min (IQR 13–55), median door-to-needle time of 46 min (IQR 35–57), and median onset-to-needle time of 139 min (IQR 92–188); symptomatic intracranial hemorrhage occurred in 4 of 51 thrombolyzed patients (7.8%).

At hospital discharge, 158 patients (50.3%) achieved a favorable functional outcome (modified Rankin Scale [mRS] 0–2), 140 (44.6%) had an unfavorable outcome (mRS 3–5), and in-hospital mortality was 5.1% (16 patients). Most patients were discharged home (237, 75.5%), while 55 (17.5%) were transferred to another facility and 6 (1.9%) were discharged against medical advice. The relatively high proportion of unfavorable outcomes reflects assessment at discharge rather than at a later, recovery-adjusted timepoint.

### Comparisons by nationality and stroke subtype

The comparison of South Asian with non–South Asian patients is shown in Table 3. The groups differed principally in demographic rather than metabolic terms: South Asian patients were far more often male and more often presented with ischemic stroke, whereas differences in hypertension, body-mass index, and dyslipidemia were not significant. The trend toward higher diabetes in South Asians did not reach significance, and ischemic heart disease was significantly less frequent.

**Table 3:** Comparison of demographic and vascular risk factors between South Asian and non–South Asian patients.

Characteristic	South Asian (*n* = 206)	Non–South Asian (*n* = 108)	*p*
Male sex	194 (94.2)	61 (56.5)	<0.001
Hypertension^c^	167 (81.1)	81 (75.0)	0.244
Diabetes mellitus	75 (36.4)	28 (25.9)	0.076
Known dyslipidemia (history)	27 (13.1)	23 (21.3)	0.074
Ischemic heart disease	6 (2.9)	9 (8.3)	0.048
BMI, kg/m^2^, median^a^	26.7	27.0	0.239
Ischemic stroke subtype^b^	144 (69.9)	58 (53.7)	0.006

Values are n (%) unless otherwise stated. p from Fisher’s exact test (pre-specified, no multiplicity correction). Comparisons unadjusted; South Asian patients were 94.2% male. BMI, body mass index.

a BMI by Mann–Whitney U; all continuous variables were non-normally distributed.

b Proportion presenting with ischemic stroke; overall subtype distribution *p* = 0.006.

c Hypertension is the composite variable (documented history or admission BP ≥140/90 mmHg).

### Factors independently associated with ischemic stroke

In the pre-specified multivariable model (Table 4), diabetes was the only factor independently associated with ischemic stroke (adjusted OR 2.34, *p* = 0.003). South Asian origin was not independently associated with ischemic presentation after adjustment, indicating that the unadjusted nationality difference was largely explained by demographic and metabolic factors; hypertension, sex, and age were not independently associated with ischemic stroke once all factors were considered together.

**Table 4:** Multivariable logistic regression: factors independently associated with ischemic (vs non-ischemic) stroke.

Predictor	Adjusted OR	95% CI	*p*
Diabetes mellitus (yes vs. no)	2.34	1.35–4.07	0.003
Hypertension (yes vs. no)^a^	0.96	0.51–1.78	0.886
South Asian origin (vs other)	1.62	0.93–2.82	0.089
Female sex (vs male)	0.69	0.35–1.35	0.277
Age (per year)	1.01	0.97–1.05	0.682

Outcome = ischemic stroke (*n* = 202) vs non-ischemic (ICH, SAH, CVST; *n* = 112). All predictors entered simultaneously. Model χ^2^ = 20.0, df = 5, *p* = 0.001; McFadden R^2^ = 0.049; area under the ROC curve = 0.65; *N* = 314. CI, confidence interval; OR, odds ratio; ROC, receiver operating characteristic.

a Hypertension defined as the composite (documented history or admission blood pressure ≥140/90 mmHg).

In the sensitivity analysis restricted to ischemic stroke and intracerebral hemorrhage (*n* = 279), every principal finding was preserved (Supplementary Table S3). The gap between documented and measured risk-factor burden was unchanged or slightly wider, small-vessel occlusion remained at 27.2% of ischemic strokes, and the independent association between diabetes and ischemic presentation strengthened (adjusted OR 2.67, 95% CI 1.41 to 5.06, *p* = 0.003). Hypertension, which showed no association in the full model, became strongly inversely associated with ischemic presentation once the comparison was confined to intracerebral hemorrhage (adjusted OR 0.21, 95% CI 0.07 to 0.61, *p* = 0.004).

### Age-band differences

Comparing patients aged 40 years or younger with those aged 41–49, vascular risk accumulated with age even within this young cohort: hypertension (68.7% vs. 86.3%, *p* < 0.001) and diabetes (24.4% vs. 38.8%, *p* = 0.008) were significantly more frequent in the older band, while subarachnoid hemorrhage and cerebral venous sinus thrombosis were proportionally more frequent in the younger band, although the overall subtype distribution did not reach significance (*p* = 0.053). Dyslipidemia and sex did not differ. The full age-band comparison is provided in Supplementary Table S2.

## Discussion

In this single-center cohort of 314 young adults with stroke from the northern UAE, the central finding is that stroke was frequently the first point at which a substantial, objectively measurable cardiometabolic burden became visible to the health system. The recorded prevalence of vascular risk factors markedly understated the burden actually present on admission, indicating widespread under-diagnosis in a young, predominantly male, South Asian migrant workforce. Three further findings characterize this burden: hypertensive and diabetic mechanisms partitioned cleanly by stroke subtype; small-vessel occlusion predominated among determined ischemic strokes, a pattern closer to young Asian cohorts than to Western registries; and nationality shaped who was exposed but did not, after adjustment, independently determine the risk profile, whereas diabetes did.

### Risk-factor burden and the UAE population context

The vascular risk-factor burden in our cohort was high for such young patients (Table 1). These figures considerably exceed general-population estimates for UAE residents. The UAE National Diabetes and Lifestyle Study, whose expatriate sample is demographically close to ours (81% male, mean age 38 years, 71% of South/Southeast Asian origin), reported a diabetes prevalence of 15.5%, hypertension 31.8%, and hypercholesterolemia 51.7% (16, 17). The roughly two-fold higher prevalence of diabetes in our stroke cohort, relative to this background population, is consistent with these patients being a vascular-risk-enriched subgroup, although this is a descriptive contrast between a hospital case series and a population survey of differing age structure and ascertainment, not a formal statistical comparison, and no inferential test was applied.

This burden sits within a broader regional trajectory: the Gulf states have undergone rapid economic and lifestyle transition, with rising obesity, sedentary behavior and dietary change driving steep increases in diabetes, hypertension and cardiovascular disease (18, 19). Population projections place the UAE among the countries most affected by obesity, and sedentary lifestyle has been specifically implicated in the regional rise of cardiometabolic disease (18). Against this backdrop, a young migrant workforce, often with limited access to preventive care and undergoing the same lifestyle transition, is plausibly predisposed to premature cerebrovascular disease. Our findings are consistent with a recent MENA systematic review reporting a high regional burden of obesity, diabetes, hypertension, and dyslipidemia among young adults, with ischemic stroke predominating at roughly four-fifths of young-stroke cases (8).

### Comparison with regional and international young-stroke cohorts

Our cohort can be situated against both regional and international young-stroke series, and the contrasts are instructive on three axes: age at presentation, subtype distribution, and risk-factor burden.

The closest demographic comparator is the BRAINS-Qatar cohort of young South Asian male migrants to the Gulf, a population structurally similar to ours in age, sex, and migration status (20). The most direct regional comparison is the 16-center MENA series of 3,971 young patients reported by Al Hashmi and colleagues, and the correspondence is close: ischemic stroke accounted for 63.6% of their cases against 64.3% of ours, with intracerebral hemorrhage 19.2% against 24.5%, cerebral venous sinus thrombosis 10.4% against 6.4%, and subarachnoid hemorrhage 7.9% against 4.8% (11). Our subtype distribution is therefore typical of clinical young-stroke series in the region, and the modest excess of intracerebral hemorrhage with correspondingly fewer venous and subarachnoid events is consistent with the referral structure described in the limitations. Our proportion of undetermined ischemic etiology (40.1%) exceeds the 22.2% reported in that series, which we attribute to the conservative adjudication described in the methods rather than to a genuinely more cryptogenic case mix.

A second UAE dataset corroborates the under-diagnosis we describe. A tertiary Abu Dhabi young-stroke series recorded 35.3% of patients as known hypertensive and 23.5% as known diabetic before admission, yet identified substantially higher rates of elevated blood pressure and abnormal glycemia on in-hospital assessment, and concluded with a call for routine screening in younger adults (12). That an independent center in a different emirate, using a different cohort and design, reproduces the same discrepancy suggests the phenomenon is a feature of the health system rather than of our institution or our ascertainment.

The comparison that matters most, however, is not of proportions but of ascertainment. Western registries draw on populations with routine primary-care contact, in which hypertension and dyslipidemia are typically identified long before a cerebrovascular event. The defining feature of our cohort is that this prior contact was largely absent, so that the risk factors driving these strokes were measurable at admission but had never been recorded. Subtype distributions can therefore be compared across settings with reasonable confidence, whereas documented risk-factor prevalences cannot, a caution that applies to any comparison between our figures and those from cohorts with established preventive care.

### Systematic under-diagnosis of vascular risk factors and the case for better primary care

The most consequential finding is the consistent gap between documented and actual vascular risk-factor burden, pointing to systematic under-diagnosis. The pattern is striking across every cardiometabolic risk factor examined. Documented hypertension was present in 49.0% of patients, yet 74.5% had elevated blood pressure on admission. Documented dyslipidemia was recorded in only 15.9%, yet an abnormal lipid profile was measured in 73.9% of those tested, a more than four-fold discrepancy. Even diabetes, the best-ascertained of the three, was previously undiagnosed in 30 of 103 affected patients (29%), each identified only because an admission HbA1c confirmed long-standing hyperglycemia.

We caution against interpreting the “known history” figures as true prevalence: they represent what had been previously diagnosed, not what was present. The true burden is better approximated by the admission measurements. While the acute stroke and its stress response can transiently elevate blood pressure and perturb lipid values, so that admission readings should not be equated uncritically with chronic disease, the magnitude and consistency of the gap cannot be explained by acute-phase effects alone, and HbA1c, which reflects months of glycaemia, is not subject to this confounding at all. The most parsimonious explanation is that a large fraction of this young, predominantly migrant population was living with untreated, undiagnosed hypertension, dyslipidemia, and diabetes until stroke became the presenting manifestation of that unmanaged risk.

The clinical and public-health implication is clear. These are young, economically active expatriate workers, most of whom have limited routine contact with primary care, often lack comprehensive insurance, and undergo infrequent health screening. The result is a population in which eminently treatable and detectable vascular risk factors go unrecognized until they cause catastrophic, disabling cerebrovascular events at a young age. Our data make a strong case that strengthening accessible primary care, workplace health screening, and affordable risk-factor management for migrant workers in the Gulf could prevent a substantial share of these strokes. Stroke in this setting is, to a considerable degree, the visible end-point of an invisible and under-treated cardiometabolic epidemic, and the most effective intervention lies upstream, in detection and control of risk factors long before the cerebrovascular event.

### Etiologic profile: a small-vessel–predominant, Asian-type pattern

Among determined ischemic strokes, small-vessel occlusion was the leading subtype (Table 2) (1, 2, 4). This is notably higher than in Mediterranean and Northern European young-stroke registries, the Athens Young Stroke Registry reported small-vessel disease in 17.4% and the Helsinki registry comparable figures, and aligns instead with the higher small-vessel proportions described in young Asian cohorts. This accords with the international BRAINS study, in which small-vessel occlusion was approximately 1.5-fold more frequent among British South Asians than in White British stroke patients, and in which South Asians experienced ischemic stroke 7 to 19 years earlier (21). The Athens investigators noted their small-vessel proportion was comparable only to data from young Asians; ours displays that pattern (1). The prominence of small-vessel disease is mechanistically consistent with the high hypertensive and diabetic burden we observed, since both are principal drivers of cerebral small-vessel pathology.

The elevated small-vessel proportion reported among British South Asians in BRAINS is most plausibly a consequence of that group’s higher cardiometabolic burden rather than of an inherited arteriopathy, and our data support that reading. Small-vessel predominance is not simply the South Asian pattern transplanted to the Gulf: young-stroke series from India report large-artery atherosclerosis and undetermined etiology as the leading subtypes, and the largest prospective Indian registry, comprising 2,066 patients across five academic centers, found small-artery occlusion in 14.2% against large-artery atherosclerosis in 29.9%, although that cohort was of all ages (mean 58.3 years) and is therefore an imperfect comparator (22). Had our patients carried an inherited predisposition to small-vessel disease with them, we would expect our distribution to resemble that of their countries of origin, and it does not. The more parsimonious explanation is exposure rather than inheritance: years of untreated hypertension and diabetes accumulated during working life in the Gulf and undetected until the index event, producing precisely the arteriolar pathology that small-vessel occlusion and deep intracerebral hemorrhage represent. On this reading the subtype distribution is a marker not of ethnicity but of the duration of unmanaged vascular risk, a pattern also evident in the geographic variation described by worldwide meta-analysis of young ischemic stroke (23).

The clean partition of risk factors by subtype reinforces this interpretation. Hypertension dominated intracerebral hemorrhage and was near-absent in cerebral venous sinus thrombosis, while diabetes concentrated in ischemic stroke (Table 1). The absence of cerebral amyloid angiopathy and medication-related hemorrhage in our ICH cases is expected given the young age and is consistent with hypertensive small-vessel rupture as the leading hemorrhagic mechanism in this medically managed cohort. However, the referral of surgical and structural hemorrhages to a partner facility (see limitations) means our ICH series under-represents structurally caused bleeds, and therefore over-represents hypertensive etiology relative to the full population. The age-band analysis further showed that this cardiometabolic burden accumulates even across the narrow 18–49 window, with venous and subarachnoid events concentrating in the youngest, supporting the view that young stroke spans biologically distinct subgroups.

The anatomical distribution of intracerebral hemorrhage in our cohort, deep in 70.1% against lobar in 14.3%, corresponds to the pattern classically associated with hypertensive arteriopathy, in which rupture occurs at the deep perforating vessels of the basal ganglia and thalamus rather than at the lobar sites more typical of amyloid angiopathy in older patients (24). That 87.0% of hemorrhages were attributable to hypertension under SMASH-U, at a median age of 42 years, is a direct expression of the under-diagnosis we describe: these were young patients whose hypertension was severe enough to rupture a perforating artery and yet had, in half of cases, never been recorded as hypertensive.

The prognostic weight of this finding is considerable. Hemorrhage volume together with level of consciousness at presentation remains the dominant determinant of 30-day mortality after intracerebral hemorrhage, and deep hemorrhages of even modest volume carry substantial risk (24). Population-based data indicate that the incidence of intracerebral hemorrhage has not declined over recent decades and that case fatality at 1 month has remained close to 40%, with no consistent improvement over time (25). In a working-age migrant population without routine access to blood-pressure monitoring, the implication is that the most lethal stroke subtype in this cohort is also the one most amenable to primary prevention by the simplest of interventions. This regional trajectory is consistent with Gulf-wide burden estimates over the same period (26).

### Nationality as setting, not independent driver

Although our cohort was predominantly South Asian, nationality did not independently determine the stroke profile. South Asian patients differed from others chiefly by being overwhelmingly male and more often ischemic, a demographic signature of the labor-migrant population, rather than by an independent excess of metabolic risk (Table 3). The trend toward higher diabetes did not reach significance, dyslipidemia was if anything lower, and in multivariable analysis South Asian origin was not independently associated with ischemic presentation once sex and metabolic factors were accounted for (Table 4). By contrast, diabetes remained an independent predictor. This pattern parallels the Saudi cohort of Eltemamy and colleagues, in which diabetes was the strongest predictor of stroke in the older young-adult stratum (27). The interpretation we favor is that ethnicity defines who is exposed in this setting, a young, male, migrant workforce, while diabetes and hypertension are the proximate mechanisms operating within that population. This framing is reinforced by the BRAINS analysis of young South Asian male migrant workers with ischemic stroke in neighboring Qatar, the demographic closest to our own cohort, which likewise identified a young, male, migrant workforce carrying a substantial modifiable vascular burden (20).

### Environmental and occupational triggers

This cohort invites consideration of environmental and occupational exposures seldom addressed in young-stroke registries from temperate, higher-income settings. Most of our South Asian patients are blue-collar laborers working outdoors in extreme Gulf heat. Chronic heat exposure with inadequate hydration promotes dehydration and hemoconcentration, raising blood viscosity and facilitating platelet activation, a plausibly pro-thrombotic milieu that may precipitate acute ischemic and venous events (28, 29); the high proportion of cerebral venous sinus thrombosis in our cohort is consistent with such a tendency, although causation cannot be established here. The psychosocial circumstances of migrant labor—long hours, low wages, family separation, remittance pressure—constitute chronic stress associated with early-onset hypertension and accelerated vascular disease (12, 30). Our dataset does not capture these exposures directly, but they offer a coherent framework for the profile observed and merit explicit measurement in future prospective Gulf cohorts.

### Diagnostic workup, the undetermined fraction, and healthcare barriers

Forty per cent of ischemic strokes were classified as undetermined, but the sub-classification (Table 2) shows this largely reflects incomplete investigation rather than true cryptogenic stroke: incomplete cardiac and incomplete multiple workup together accounted for the majority, and echocardiography was performed in only a third of the cohort. These gaps are not solely methodological; in this setting they are driven substantially by socioeconomic and systemic barriers characteristic of blue-collar migrant healthcare: out-of-pocket costs and limited insurance coverage that deter elective investigations, restricted availability of specialized tests such as echocardiography and advanced vascular or thrombophilia studies outside of weekday hours, and early discharge or rapid repatriation of expatriate workers, sometimes following job loss precipitated by the stroke itself, before a full workup can be completed (10). Viewed this way, the high undetermined fraction is not merely a limitation of the present analysis but an informative signal of the systemic obstacles facing young migrant workers with stroke in the Gulf, and a concrete target for service improvement. We intend to examine the determinants and consequences of incomplete workup in detail in a separate analysis.

### Strengths and limitations

The principal strengths of this study are its consistent, single-adjudicator classification applied uniformly across the full spectrum of cerebrovascular disease, its inclusion of hemorrhagic and venous events alongside ischemic stroke, and its transparent handling of exclusions and missing data. Several limitations should be acknowledged. First, the retrospective single-center design limits generalizability and introduces documentation gaps, including incomplete prior diagnostic labeling that required us to supplement historical hypertension and dyslipidemia diagnoses with admission measurements; the admission blood-pressure and lipid variables, conversely, may be transiently influenced by the acute stress response of stroke and should be read as reflecting the state at presentation rather than necessarily the chronic baseline. Second, anthropometric assessment relied on body-mass index alone, without waist circumference or other measures of central adiposity. This is an important limitation in a predominantly South Asian cohort, because South Asians characteristically exhibit a “thin-fat” or sarcopenic-obese phenotype, with greater visceral adiposity and insulin resistance at any given body-mass index than other populations (31, 32). Regional Gulf data indicate that ethnicity-specific anthropometric cut-points are required to discriminate diabetes and the metabolic syndrome in South Asians and Arabs, since standard thresholds misclassify cardiometabolic risk in these groups (33). Standard thresholds therefore probably underestimate the true cardiometabolic risk here. Third, functional outcome was assessed at discharge rather than at 90 days, which overstates disability relative to recovery-adjusted timepoints, and longitudinal follow-up was not available. Fourth, the high undetermined fraction reflects incomplete diagnostic workup, itself shaped by the socioeconomic barriers discussed above. Fifth, small subgroup sizes for SAH and CVST limit the precision of subtype-specific estimates. Sixth, and importantly, because IBHOH is a medical hospital without on-site neurosurgical or neurovascular services, patients requiring surgical or interventional management are referred to a partner surgical facility (Saqr Hospital). As a result, surgically managed hemorrhagic strokes, in particular aneurysmal subarachnoid hemorrhage and structural intracerebral hemorrhage (vascular malformations, tumors, and aneurysmal causes), are likely under-represented in this cohort. The number of SAH cases should therefore be regarded as a minimum, and the predominance of hypertensive etiology among our intracerebral hemorrhages partly reflects the medically managed nature of the admitted population, rather than the full causal spectrum of young hemorrhagic stroke in the region. Finally, the comparison of risk-factor prevalence with general-population figures is descriptive only and should not be read as a formal case–control inference.

A further limitation concerns the nationality comparison. The non-South Asian comparator group is highly heterogeneous, comprising 108 patients of 21 nationalities, of which the largest accounted for only 17.6% of the group and nine contributed two patients or fewer. Such heterogeneity may introduce confounding and limits the interpretability of the nationality contrast. We considered further subgroup analysis but concluded that the cell counts do not support it, since no individual non-South Asian nationality reaches a size at which stratified risk-factor comparison would be meaningful; the four-group regional stratification presented in Supplementary Table S1 is the finest division the data will bear. This limitation does not bear on the central finding of the study, which concerns the gap between documented and measured risk-factor burden across the cohort as a whole and does not depend on the ethnic contrast, and South Asian origin was not independently associated with ischemic presentation after adjustment.

## Conclusion

Young stroke in this predominantly South Asian Gulf cohort is characterized by a high, subtype-differentiated burden of hypertensive and diabetic cerebrovascular disease, with a small-vessel–predominant ischemic pattern resembling young Asian populations more than Western ones. Diabetes was the only factor independently associated with ischemic stroke, while nationality shaped the exposed population without independently determining the risk profile. A central message of this study is that the true burden of vascular risk factors in this population is substantially greater than prior medical records suggest: across hypertension, dyslipidemia, and diabetes, conditions detected by objective admission measurement far outnumbered those previously documented, indicating widespread under-diagnosis in a young migrant workforce with limited access to routine care. The low recorded prevalence of these risk factors should therefore not be mistaken for low true prevalence. These findings point to a concrete and actionable priority: strengthening accessible primary care, workplace screening, and affordable risk-factor management for expatriate workers, so that treatable cardiometabolic disease is detected and controlled before it presents as disabling stroke in young adulthood. Larger, prospective, multicenter Gulf registries are needed to confirm these patterns and to define region-specific prevention strategies for this young and economically vital population.

## Data Availability

The raw data supporting the conclusions of this article will be made available by the authors, without undue reservation.
