# Supplementary material for: The silent risk: cardiometabolic burden and etiology of young stroke in a South Asian migrant cohort, United Arab Emirates
Source: Front Neurol. 2026 Sep 17;17:1940843. doi: 10.3389/fneur.2026.1940843 (PMC13627398; doi:10.3389/fneur.2026.1940843)
Supplement: Supplementary file 1 [file Table_1.DOCX]

# **Supplementary Materials**

*The Silent Risk: Cardiometabolic Burden and Etiology of Young Stroke in a South Asian Migrant Cohort, United Arab Emirates*

**Supplementary Table S1. Demographic, subtype, and vascular risk-factor profile by nationality / region of origin.**

| **Characteristic** | **South Asian (n=206)** | **MENA (n=34)** | **Other (n=62)** | **Emirati (n=12)** |
| --- | --- | --- | --- | --- |
| Demographics |  |  |  |  |
| Age, years, mean ± SD | 41.5 ± 5.5 | 39.7 ± 7.1 | 40.4 ± 6.5 | 39.3 ± 6.2 |
| Male sex | 194 (94.2) | 24 (70.6) | 28 (45.2) | 9 (75.0) |
| BMI, kg/m², median | 26.7 | 27.5 | 26.0 | 31.0 |
| Stroke subtype |  |  |  |  |
| Ischemic stroke (AIS) | 144 (69.9) | 20 (58.8) | 31 (50.0) | 7 (58.3) |
| Intracerebral hemorrhage (ICH) | 48 (23.3) | 4 (11.8) | 22 (35.5) | 3 (25.0) |
| Subarachnoid hemorrhage (SAH) | 6 (2.9) | 4 (11.8) | 5 (8.1) | 0 (0) |
| Cerebral venous sinus thrombosis (CVST) | 8 (3.9) | 6 (17.6) | 4 (6.5) | 2 (16.7) |
| Vascular risk factors |  |  |  |  |
| Hypertension ᵃ | 167 (81.1) | 25 (73.5) | 48 (77.4) | 8 (66.7) |
| Diabetes mellitus | 75 (36.4) | 9 (26.5) | 13 (21.0) | 6 (50.0) |
| Known dyslipidemia (history) | 27 (13.1) | 10 (29.4) | 7 (11.3) | 6 (50.0) |

Values are n (%) unless otherwise stated. Emirati proportions (n = 12) should be interpreted with caution given the small cell count. South Asian comprises India, Pakistan, Bangladesh, Sri Lanka, Nepal, and Afghanistan. ᵃ Hypertension is the composite variable (documented history or admission blood pressure ≥140/90 mmHg). AIS, acute ischemic stroke; BMI, body mass index; CVST, cerebral venous sinus thrombosis; ICH, intracerebral hemorrhage; MENA, Middle East and North Africa; SAH, subarachnoid hemorrhage; SD, standard deviation.

**Supplementary Table S2. Vascular risk factors and stroke subtype by age band.**

| **Characteristic** | **≤40 years (n=131)** | **41–49 years (n=183)** | **p** |
| --- | --- | --- | --- |
| Demographics |  |  |  |
| Male sex | 111 (84.7) | 144 (78.7) | 0.189 |
| Vascular risk factors |  |  |  |
| Hypertension ᵃ | 90 (68.7) | 158 (86.3) | <0.001 |
| Diabetes mellitus | 32 (24.4) | 71 (38.8) | 0.008 |
| Known dyslipidemia (history) | 17 (13.0) | 33 (18.0) | 0.274 |
| Stroke subtype |  |  |  |
| Ischemic stroke (AIS) | 77 (58.8) | 125 (68.3) |  |
| Intracerebral hemorrhage (ICH) | 32 (24.4) | 45 (24.6) |  |
| Subarachnoid hemorrhage (SAH) | 10 (7.6) | 5 (2.7) |  |
| Cerebral venous sinus thrombosis (CVST) | 12 (9.2) | 8 (4.4) | 0.053 |

Values are n (%). p from Fisher's exact test; the single p-value for stroke subtype refers to the overall distribution across the four subtypes. ᵃ Hypertension is the composite variable (documented history or admission blood pressure ≥140/90 mmHg). AIS, acute ischemic stroke; CVST, cerebral venous sinus thrombosis; ICH, intracerebral hemorrhage; SAH, subarachnoid hemorrhage.

**S3a. Sensitivity analysis: principal findings with subarachnoid hemorrhage and cerebral venous sinus thrombosis excluded.**

| **Characteristic** | **Full cohort (n=314)** | **Restricted (n=279)** | **Change** |
| --- | --- | --- | --- |
| Documented hypertension | 154 (49.0) | 148 (53.0) | +4.0 |
| Elevated BP on admission | 234 (74.5) | 222 (79.6) | +5.1 |
| Hypertension, composite | 248 (79.0) | 234 (83.9) | +4.9 |
| Documented dyslipidemia | 50 (15.9) | 47 (16.8) | +0.9 |
| Abnormal lipid profile, of tested | 184/249 (73.9) | 170/231 (73.6) | −0.3 |
| Diabetes mellitus | 103 (32.8) | 96 (34.4) | +1.6 |
| Diabetes newly diagnosed, of diabetic | 30/103 (29.1) | 25/96 (26.0) | −3.1 |
| Small-vessel occlusion, of AIS | 55/202 (27.2) | 55/202 (27.2) | 0.0 |

Values are n (%) unless otherwise stated. Change is expressed in percentage points. The restricted cohort comprises acute ischemic stroke and intracerebral hemorrhage only (15 SAH and 20 CVST excluded). AIS, acute ischemic stroke; BP, blood pressure.

**S3b. Multivariable logistic regression for ischemic versus non-ischemic stroke, full and restricted cohorts.**

| **Predictor** | **Full aOR** | **95% CI** | **Restricted aOR** | **95% CI** |
| --- | --- | --- | --- | --- |
| Diabetes mellitus | 2.34 | 1.35–4.07 | 2.67 | 1.41–5.06 |
| Hypertension, composite | 0.95 | 0.51–1.77 | 0.21 | 0.07–0.61 |
| South Asian origin | 1.59 | 0.91–2.76 | 1.31 | 0.69–2.50 |
| Male sex | 1.55 | 0.79–3.04 | 1.55 | 0.70–3.45 |
| Age, per year | 1.01 | 0.97–1.05 | 1.01 | 0.96–1.06 |

In the restricted cohort the comparison is ischemic stroke versus intracerebral hemorrhage. Diabetes remains independently associated with ischemic presentation (p = 0.003 in both models). Hypertension becomes strongly inversely associated with ischemic presentation (p = 0.004) once the pooled non-ischemic category is confined to intracerebral hemorrhage, consistent with the SMASH-U attribution of 87.0% of hemorrhages to hypertension. aOR, adjusted odds ratio.

**S3c. Anatomical and etiological distribution of intracerebral hemorrhage (n = 77).**

| **Anatomical location** | **n** | **%** | **SMASH-U category** | **n (%)** |
| --- | --- | --- | --- | --- |
| Deep — putamen | 37 | 48.1 | Hypertension | 67 (87.0) |
| Deep — thalamus | 15 | 19.5 | Undetermined | 7 (9.1) |
| Deep — caudate | 2 | 2.6 | Structural lesion | 2 (2.6) |
| Infratentorial — brainstem | 9 | 11.7 | Systemic/other | 1 (1.3) |
| Infratentorial — cerebellum | 3 | 3.9 | — | — |
| Lobar — unspecified | 7 | 9.1 | — | — |
| Lobar — temporal | 4 | 5.2 | — | — |

Grouped: deep 54 (70.1%), infratentorial 12 (15.6%), lobar 11 (14.3%). Subarachnoid hemorrhage (n = 15) was classified separately as aneurysmal (n = 12) or non-aneurysmal (n = 3) and was not subjected to SMASH-U. SMASH-U, structural lesion / medication / amyloid angiopathy / systemic disease / hypertension / undetermined.

**S3d. National composition of the non-South Asian comparator group (n = 108).**

| **Nationality** | **n** | **%** | **Nationality** | **n** | **%** |
| --- | --- | --- | --- | --- | --- |
| Filipino | 19 | 17.6 | Iraqi | 4 | 3.7 |
| Indonesian | 14 | 13.0 | Ethiopian | 3 | 2.8 |
| Emirati | 12 | 11.1 | Yemeni | 2 | 1.9 |
| Egyptian | 10 | 9.3 | Moroccan | 2 | 1.9 |
| Nigerian | 8 | 7.4 | Russian | 2 | 1.9 |
| Ghanaian | 6 | 5.6 | Palestinian | 1 | 0.9 |
| Jordanian | 5 | 4.6 | Sierra Leonean | 1 | 0.9 |
| Comoran | 5 | 4.6 | Chinese | 1 | 0.9 |
| Sudanese | 5 | 4.6 | Ugandan | 1 | 0.9 |
| Omani | 5 | 4.6 | American | 1 | 0.9 |
|  |  |  | Myanmar | 1 | 0.9 |

The comparator group comprises 21 nationalities; no single nationality exceeds 17.6% of the group. For reference, the South Asian group (n = 206) comprises Indian (99), Bangladeshi (57), Pakistani (41), Nepalese (4), Afghan (3) and Sri Lankan (2).

**S3e. Shapiro–Wilk tests of normality (n = 314).**

| **Variable** | **n** | **W** | **p** | **Median (IQR)** |
| --- | --- | --- | --- | --- |
| Age, years | 314 | 0.943 | <0.001 | 42 (38–46) |
| NIHSS | 310 | 0.720 | <0.001 | 5 (2–11) |
| Systolic BP, mmHg | 314 | 0.975 | <0.001 | 160 (134–185) |
| Diastolic BP, mmHg | 314 | 0.981 | <0.001 | 98 (83–115) |
| Glucose, mmol/L | 314 | 0.822 | <0.001 | 7.4 (6.0–10.7) |
| BMI, kg/m² | 296 | 0.945 | <0.001 | 27.0 (24.0–30.0) |
| Symptom duration, h | 310 | 0.503 | <0.001 | 5.3 (2.0–23.5) |
| mRS at discharge | 314 | 0.938 | <0.001 | 2 (1–4) |

All continuous variables departed significantly from normality; non-parametric tests were therefore applied throughout. BMI and NIHSS were missing in 18 and 4 patients respectively. BMI, body mass index; IQR, interquartile range; mRS, modified Rankin Scale; NIHSS, National Institutes of Health Stroke Scale.
